# Osthole, a Natural Plant Derivative Inhibits MRGPRX2 Induced Mast Cell Responses

**DOI:** 10.3389/fimmu.2020.00703

**Published:** 2020-04-24

**Authors:** Brianna N. Callahan, Ananth K. Kammala, Meesum Syed, Canchai Yang, Christopher J. Occhiuto, Rithvik Nellutla, Alena P. Chumanevich, Carole A. Oskeritzian, Rupali Das, Hariharan Subramanian

**Affiliations:** ^1^Department of Physiology, Michigan State University, East Lansing, MI, United States; ^2^Department of Pathology, Microbiology, and Immunology, School of Medicine, University of South Carolina, Columbia, SC, United States

**Keywords:** mast cells, pseudo-allergic reactions, osthole, MAS-related G-protein coupled receptor-X2, MrgprB2

## Abstract

Mast cells are tissue-resident innate immune cells known for their prominent role in mediating allergic reactions. MAS-related G-protein coupled receptor-X2 (MRGPRX2) is a promiscuous G-protein coupled receptor (GPCR) expressed on mast cells that is activated by several ligands that share cationic and amphipathic properties. Interestingly, MRGPRX2 ligands include certain FDA-approved drugs, antimicrobial peptides, and neuropeptides. Consequently, this receptor has been implicated in causing mast cell-dependent pseudo-allergic reactions to these drugs and chronic inflammation associated with asthma, urticaria and rosacea in humans. In the current study we examined the role of osthole, a natural plant coumarin, in regulating mast cell responses when activated by the MRGPRX2 ligands, including compound 48/80, the neuropeptide substance P, and the cathelicidin LL-37. We demonstrate that osthole attenuates both the early (Ca^2+^ mobilization and degranulation) and delayed events (chemokine/cytokine production) of mast cell activation via MRGPRX2 *in vitro*. Osthole also inhibits MrgprB2- (mouse ortholog of human MRGPRX2) dependent inflammation in *in vivo* mouse models of pseudo-allergy. Molecular docking analysis suggests that osthole does not compete with the MRGPRX2 ligands for interaction with the receptor, but rather regulates MRGPRX2 activation via allosteric modifications. Furthermore, flow cytometry and confocal microscopy experiments reveal that osthole reduces both surface and intracellular expression levels of MRGPRX2 in mast cells. Collectively, our data demonstrate that osthole inhibits MRGPRX2/MrgprB2-induced mast cell responses and provides a rationale for the use of this natural compound as a safer alternative treatment for pseudo-allergic reactions in humans.

## Introduction

Mast cells are innate immune cells that are best known for their role in causing allergic reactions. Crosslinking of the high-affinity IgE receptor (FcεRI) on mast cells by the IgE-allergen complex results in the rapid release of inflammatory mediators that triggers allergy. Mast cells are also activated through IgE-independent pathways via G-protein coupled receptors (GPCR) such as the receptors for the complement components, C3a, and C5a (1, 2). A new development in mast cell research has been the identification of the non-canonical GPCR Mas-related G-protein coupled receptor X2 (MRGPRX2) in humans and its ortholog MrgprB2 in mice (3, 4). This receptor is expressed on connective tissue-type mast cells in the skin (5) and lungs (6). Interestingly, unlike other GPCRs, MRGPRX2 binds to multiple ligands and is potently activated by small cationic molecules and peptides that have amphipathic properties; therefore, this receptor can elicit responses to a wide variety of endogenous and exogenous ligands (7, 8). As a result, MRGPRX2 is responsible for mediating mast cell anaphylactic and pseudo-allergic reactions to several US Food and Drug Administration (FDA)-approved drugs (3, 9–14) and has been implicated in the pathology of chronic inflammatory diseases such as asthma (6), rosacea (15), and urticaria (5). Thus, current research is focused on identifying new inhibitors that can target MRGPRX2 responses and subsequently prevent mast cell activation in these diseases.

Osthole (7-methoxy-8-isopentenoxycoumarin) is a natural coumarin present in the fruits of the *Cnidium monnieri* (L.) Cusson plant. Crude extracts from the dried fruits of *Cnidium monnieri* (L.) Cusson has been extensively used as a traditional Chinese medicine to treat various conditions such as osteoporosis (16), pulmonary inflammation (17) and certain skin diseases (18, 19). Osthole is an important constituent of the dried fruits and has been recognized as a promising lead compound in drug discovery research. Osthole is known to possess a variety of pharmacological activities; including anti-inflammation (20–22), antitumor (23–26), and antidiabetic properties (27, 28). It has been reported that osthole inhibited the development of inflammatory diseases such as arthritis (29) and hepatitis (30, 31) in animal models. Matsuda et al., (32) showed that osthole has an antipruritic effect in an allergic mouse model. Interestingly, recent reports have demonstrated that osthole protects against atopic dermatitis (18, 33) and allergic asthma (34) in murine models. Additionally, Chiang et al., (35) showed that osthole treatment attenuated Th2 mediated allergic asthma by modulating dendritic cell maturation and functions. These reports highlight the therapeutic potential of osthole in treating allergic diseases; however, whether osthole regulates mast cell responses during allergic/anaphylactic reactions has not yet been examined.

In the current study, we aimed to determine the role of osthole in modulating mast cell response following activation via the MRGPRX2 (human)/MrgprB2 (murine) receptors. Given that osthole inhibited allergic responses in animal models and the mast cell-MRGPRX2 axis is essential for causing anaphylactic reactions, we hypothesized that osthole inhibits MRGPRX2/MrgprB2-mediated mast cell activation. Our data demonstrate that osthole significantly impairs human mast cell activation to the MRGPRX2 ligands compound 48/80 (3), the neuropeptide substance P (8, 36), and the cathelicidin LL-37 (37) *in vitro*. Specifically, mast cell pretreatment with osthole resulted in a robust reduction in intracellular Ca^2+^ mobilization, degranulation, cytokine (IL-8) and chemokine (MCP-1) production and activation of mitogen-activated protein (MAP) kinase pathway following MRGPRX2 stimulation. In agreement with our *in vitro* data, this natural coumarin also attenuated MrgprB2-induced mast cell responses *in vivo* in mouse models of paw edema as well as experimental rosacea. Molecular docking studies implicate that osthole does not directly compete with the MRGPRX2 ligands for interaction with the receptor. Additionally, our studies reveal that osthole modulates mast cell activation via regulation of MRGPRX2 expression. Taken together, we demonstrate for the first time that osthole inhibits MRGPRX2/MrgprB2 responses in mast cells. This plant-derived coumarin can thus be clinically exploited for treatment of anaphylactic and/or pseudo-allergic reactions in humans.

## Materials and Methods

### Tissue Culture Media and Reagents

Dulbecco’s Modified Eagle’s Media (DMEM), Iscove’s Modified Dulbecco’s Media (IMDM), penicillin, streptomycin and L-glutamine supplement were purchased from Corning Cellgro^TM^ (Corning, NY, United States). Recombinant human stem cell factor (hSCF) was purchased from PeproTech (Rocky Hill, NJ, United States). Opti-MEM^TM^, Stem-Pro^TM^-34 SFM media, and TRIzol^TM^ were purchased from Invitrogen (Carlsbad, CA, United States). Chemical reagents used in buffers, unless otherwise noted, were purchased from Sigma-Aldrich (St. Louis, MO, United States). Compound 48/80, substance P and (*R*)-ZINC-3573 were obtained from Sigma-Aldrich. Cathelicidin LL-37 (Leu-Leu-Gly-Asp-Phe-Phe-Arg-Lys-Ser-Lys-Glu-Lys-Ile-Gly-Lys-Glu-Phe-Lys-Arg-Ile-Val-Gln-Arg-Ile-Lys-Asp-Phe-Leu-Arg-Asn-Leu-Val-Pro-Arg-Thr-Glu-Ser) was purchased from Tocris Bioscience (Minneapolis, MN, United States). Osthole was purchased from Cayman Chemicals (Ann Arbor, MI, United States). The initial stock solution of osthole was made in DMSO, but subsequent dilutions were performed in phosphate-buffered saline (PBS). All kits for cDNA synthesis, TaqMan^TM^ probes and quantitative PCR were obtained from Applied Biosystems (Foster City, CA, United States). ELISA kits (MCP-1 and IL-8) and MTT reagent were purchased from Invitrogen. Primary western blotting antibodies (anti-phospho-p44/42 and anti-p44/42) were obtained from Cell Signaling Technology (Danvers, MA), and secondary antibodies (donkey anti-rabbit conjugated to IRDye^®^ 680RD and IRDye^®^ 800CW) were purchased from Li-Cor Biosciences (Lincoln, NE). Anti-human MRGPRX2-PE antibody (clone: K125H4) and isotype-PE antibody (clone: poly4053) were purchased from BioLegend (San Diego, CA, United States).

### Mice

C57BL/6 and Balb/c mice were obtained from the Jackson Laboratory (Bar Harbor, ME, United States). All mice were kept under specific pathogen-free conditions. All experiments had the approval of the Institutional Animal Care and Use Committee at Michigan State University. Both male and female mice (6–8 weeks old) were used for experiments.

### Cells

Human LAD2 mast cells were cultured in complete Stem-Pro-34 SFM medium containing penicillin (100 IU/mL), streptomycin (100 μg/mL) and L-glutamine (2 mM) (PSG) supplemented with recombinant hSCF (100 ng/mL) as described previously (38). Media was hemi-depleted once every week and cells were maintained at a concentration of 0.8 × 10^6^ cells/mL. Rat basophilic leukemia (RBL-2H3) cells were obtained from the American Type Culture Collection (Manassas, VA, United States) and were cultured in DMEM supplemented with 10% bovine calf serum and PSG. Cells were split every other day. RBL-2H3 cells stably expressing MRGPRX2 were generated as described previously (37) and were cultured in DMEM supplemented with 10% fetal bovine serum, PSG and G418 (1 mg/mL). For isolation of mouse peritoneal cells, the peritoneal cavity of C57BL/6 mice was lavaged with 5 ml of IMDM supplemented with 10% bovine calf serum and cells were centrifuged, counted, and used for degranulation experiments as described below.

Human skin mast cells were isolated from 4 different donors and cultured as described previously (39, 40). Briefly, mast cells were isolated and purified from fresh surgical specimens of human skin tissues that were purchased from the Cooperative Human Tissue Network (CHTN) of the National Cancer Institute. These studies were approved by the human studies Internal Review Board (IRB) of the University of South Carolina. The tissues were mechanically minced and digested with collagenase type II, hyaluronidase and DNase I in HBSS buffer (1X HBSS, 0.04% NaHCO3, 1% fetal bovine serum, 1% HEPES, 0.1% CaCl_2_). The samples were filtered through 40 μm cell strainers and separated on a Percoll cushion by density centrifugation. The cells at the interface of buffer and Percoll layers were collected, washed and resuspended at 5 × 10^5^ cells/mL in serum-free X-VIVO 15^TM^ media (Lonza) supplemented with hSCF (100 ng/mL). They were cultured at 37°C and 5% CO_2_ with weekly media changes for 8 weeks. Purity was assessed by metachromatic staining with acidic toluidine blue and by flow cytometry staining for FcεRI expression with PE-labeled anti-human FcεRI antibody [clone AER-37 (CRA)] and mouse IgG2bk isotype control (BioLegend). The mast cells were used only when >95% of the cells were FcεRI+ (after ∼8 weeks of culture).

### Cytotoxicity Assays

LAD2 cells (1 × 10^5^/well) were plated on 96-well plates and treated with varying concentrations of osthole for 48 h. The cells were harvested and live and dead cells were counted using a hemocytometer after staining with trypan blue. For determining the viability of RBL-2H3 cells, the MTT assay was used. Briefly, RBL-2H3 cells (0.5 × 10^5^/well) were plated on 96-well plates and treated with different concentrations of osthole for 48 h. The cells were exposed to the MTT reagent (1.2 μM) for 4 h at 37°C. A total lysate sample was also included for the assays in which the cells were lysed with 10% Triton X-100 reagent. The formazan crystals formed by the MTT reagent were dissolved in 10% DMSO. Absorbance was measured using FlexStation^®^ 3 multi-mode plate reader at 540 nm and percent viability was calculated using the following formula: (O.D. value of the osthole treated sample) – (OD value of the total lysate sample) × 100.

### Calcium Mobilization

LAD2 cells were washed and resuspended in 1 mL of 0.1% SIR-BSA (118 mM NaCl, 5 mM KCl, 25 mM HEPES, 5.5 mM glucose, 0.4 mM MgCl_2_, 1 mM CaCl_2_, and 1 mg/mL bovine serum albumin) supplemented with 6 μM Fluo-8 AM calcium dye (Abcam; Cambridge, United Kingdom) for 1 h at 37°C and 5% CO_2_. Cells were washed with 0.1% SIR-BSA and 100 μL (0.3 × 10^6^ cells/mL) and seeded in the same buffer with different concentrations of osthole for 30 min. For assays using RBL-2H3 cells expressing MRGPRX2, 50 μL (1 × 10^6^ cells/mL) of cells were plated per well of a 96-well plate. The cells were then labeled with Fluo-8 AM and treated with osthole as described above. Using the FlexStation^®^ 3 Flex-protocol, changes in fluorescence (maximum - minimum values) were measured with the addition of compound 48/80, substance P, LL-37 or (*R*)-ZINC-3573. Excitation and emission wavelengths were 490 and 520 nm, respectively.

### Degranulation Assay

LAD2 cells, primary human skin-derived mast cells and mouse peritoneal cells were resuspended in 0.1% SIR-BSA containing different concentrations of osthole. After 30 min, 45 μL of cells (0.45 × 10^6^ cells/mL for LAD2 and skin mast cells and 2 × 10^6^ cells/mL for mouse peritoneal cells) were seeded per well of 96-well plate and stimulated with compound 48/80, substance P, LL-37 or (*R*)-ZINC-3573 for 30 min. For assays using RBL-2H3 cells expressing MRGPRX2, 45 μL (1.2 × 10^6^ cells/mL) of cells were plated per well of a 96-well plate and treated with different concentrations of osthole and the MRGPRX2 agonists as described above. Supernatant were collected and incubated with an equivalent volume of 4 mM p-nitrophenyl-N-acetyl-β-D-glucosamine (PNAG, Sigma-Aldrich) for 1 h at 37°C. The reactions were halted through the addition of 0.1 M NaHCO_3_/0.1M Na_2_CO_3_ buffer. Absorbance was measured using FlexStation^®^ 3 multi-mode plate reader (Molecular Devices; San Jose, CA, United States) at 405 nm. The total β-hexosaminidase content was measured by lysing cells with 0.1% Triton X-100 and then incubating the supernatant of the lysed cells with PNAG. Percent β-hexosaminidase release content was calculated by dividing the absorbance of agonist-stimulated cells by total cell β-hexosaminidase content. The spontaneous β-hexosaminidase release for LAD2, RBL-2H3 and primary human skin-derived mast cells was between 5 and 20% and for mouse peritoneal cells was between 4 and 20%.

### ELISA

LAD2 cells were washed twice in cytokine-deprived complete Stem-Pro 34 media, then 150 μL (1 × 10^6^ cells/mL, 0.15 × 10^6^ cells/well) were plated and incubated with different concentrations of osthole for 30 min and stimulated with corresponding agonists for 6 h. Cells were centrifuged, and supernatants collected. Cytokines/chemokines in the supernatants (IL-8 and MCP-1) were quantified by ELISA. The levels of cytokines in untreated samples varied between 200 and 450 ng/ml and 2–6 pg/ml for MCP-1 and IL-8, respectively.

### Western Blotting

LAD2 cells (4 × 10^6^ cells/mL) were incubated with osthole (72 μM) for 30 min, stimulated with substance P (300 nM) for different time intervals and lysed using radioimmunoprecipitation assay (RIPA) [150 mM NaCl, 1.0% Triton X-100, 0.5% sodium-deoxycholate, 0.1% sodium dodecyl sulfate, 25 mM Tris (pH 8.0), 5 mM EDTA] buffer with protease inhibitor cocktail (Roche Applied Sciences; Mannheim, Germany). A total of 30 μg of protein was loaded in a 10% polyacrylamide gel for electrophoretic separation. Proteins were then transferred to nitrocellulose membranes (GE Healthcare). Membranes were blocked in 5% milk solution for 2 h, washed in Tris-buffered saline [pH 7.6] with 0.1% Tween-20 (TBST), then probed with primary antibodies (anti-phospho-p44/42 and anti-p44/42). The following day, blots were washed in TBST and probed with LiCor IRDye^®^ 680RD or IRDye^®^ 800CW conjugated secondary antibodies for 2 h in the dark. Blots were imaged using LiCor Odyssey Imaging Systems (Lincoln, NE, United States) and analyzed using ImageJ software.

### Paw Edema Model

C57BL/6 mice were initially treated with vehicle (0.2% DMSO in PBS) or osthole (100 mg/kg) for 3 days via i.p., injection. On the fourth day, mice received PBS in the right hind paw and compound 48/80 in the left hind paw (150 ng in 5 μL). Mice were then injected retro-orbitally with 0.15% Evans blue. After 30 min, paws were excised, dried at 50°C and placed into acetone:saline (7:3). The dye was allowed to diffuse from the tissue for 48 h and the absorbance of the supernatant was quantified at 650 nm. For some experiments, the paw thickness of mice was measured before and after compound 48/80 treatment with a micrometer thickness gauge (Peacock thickness gauge, G-1A) and change in paw thickness was calculated.

### Cathelicidin LL-37-Induced Rosacea Model

Balb/c mice received i.p. injections of vehicle (0.2% DMSO in PBS) or osthole (100 mg/kg) for 4 days. Mice were then given intradermal injections (i.d.) of LL-37 (50 μL of 320 μM) on their back skin twice a day for 2 days while continuing the osthole treatment. Seventy-two hours after the last LL-37 injection, skin tissues were harvested and either snap-frozen in liquid N_2_ for RNA analysis or fixed in 10% formalin solution for H&E staining. Skin inflammation was scored as described previously by Schwartz et al. (41). Briefly, an objective scoring system was employed in a blinded fashion. Erythema, scaling and thickening were scored independently from 0 to 4 as follows: 0, none; 1, slight; 2, moderate; 3, marked; and 4, extreme. The average cumulative score of erythema, scaling and thickening served to indicate the inflammation score (scale 0–4). For epidermal thickness measurements, 5 random epidermal areas in H&E stained skin sections from each mouse were chosen and measured following the acquisition of images using a Nikon^®^ ECLIPSE 50i microscope equipped with a Lumenera^®^ Infinity 3 color camera. For assessing *in vivo* mast cell degranulation, skin tissues were stained with toluidine blue (0.1% in PBS, pH 2.3) and images were captured as described above. Degranulated mast cells (as determined by the staining intensity, appearance and/or location of the granules) were counted and expressed as percentage of total mast cells in the tissue sections (42).

### Real-Time PCR

Skin samples taken from mice were homogenized in liquid N_2_ using a mortar and pestle. RNA was extracted using TRIzol^TM^ reagent according to the manufacturer’s protocol. RNA (2 μg) was transcribed to cDNA using the high capacity cDNA reverse transcription kit from Applied Biosystems. RNA levels (*Ccl2*, *Il6*, *Tnf*, and *Mmp9*) were quantified using gene expression assays with TaqMan^TM^ Fast Advanced Master Mix and validated TaqMan^TM^ probes.

### Molecular Docking

MRGPRX2 protein structure and function were predicted using I-TASSER. The most optimal energy conformation was chosen for docking analysis. SwissDock was used to determine the optimal binding location for osthole, compound 48/80 and substance P. Potential binding sites were analyzed by lowest energy conformation and the software determined ideal intermolecular forces. The top models were chosen to conduct further docking. Molecular graphics and analyses were performed with University of California at San Francisco (UCSF) Chimera, developed by the resource for bio-computing, visualization and informatics at UCSF.

### Flow Cytometry

LAD2 cells (5 × 10^5^) were stimulated with osthole (72 μM) or vehicle (0.2% DMSO in PBS) for 5, 30, and 60 min. Cells were washed twice with FACS buffer (10% FBS in PBS) and stained with anti-human MRGPRX2-PE antibody for 30 min in the dark. Cells were then washed with FACS buffer and fixed with 1.6% paraformaldehyde. Data was collected on a LSRII flow cytometer (BD Biosciences) and MRGPRX2 expression was analyzed using FlowJo software (FlowJo LLC, Ashland, OR, United States).

### Confocal Microscopy

LAD2 cells were treated with osthole (72 μM) at different timepoints (0, 15, and 30 min). After stimulation, cells were pelleted, washed with PBS, cytospun onto slides and fixed with 4% paraformaldehyde at room temperature. The slides were then permeabilized with 0.5% saponin for 10 min and blocked at room temperature for 1 h with blocking buffer (1% bovine serum albumin). After three washes with PBS with 0.1% Tween (PBST), slides were incubated with anti-human MRGPRX2 antibody (Novus Biologicals, Centennial, CO, United States) in dilution buffer (PBS containing 1% BSA, 1:500 dilution) in a humidified chamber at room temperature for 1 h. Slides were washed 3 times with PBST and incubated with anti-rabbit Alexa Fluor 555-conjugated IgG secondary antibody (Invitrogen) in dilution buffer (1:300) for 1 h at room temperature in the dark followed by incubation in 300 μM DAPI (1:1000 dilution). Slides were then washed 3 times in PBST and mounted using the ProLong^TM^ Diamond antifade mounting media (Invitrogen). Confocal images were obtained with the Olympus FV 1000 confocal laser scanning microscope (Olympus America, Center Valley, PA, United States) and images were analyzed using the ImageJ software.

### Statistical Analysis

Statistical analyses were performed using GraphPad PRISM software (San Diego, CA, United States) and explained in the figure legends or the results sections. A *p*-value less than or equal to 0.05 was deemed to be significant.

## Results

### Osthole Attenuates MRGPRX2-Mediated Ca^2+^ Mobilization and Degranulation in Mast Cells

**FIGURE 1:**
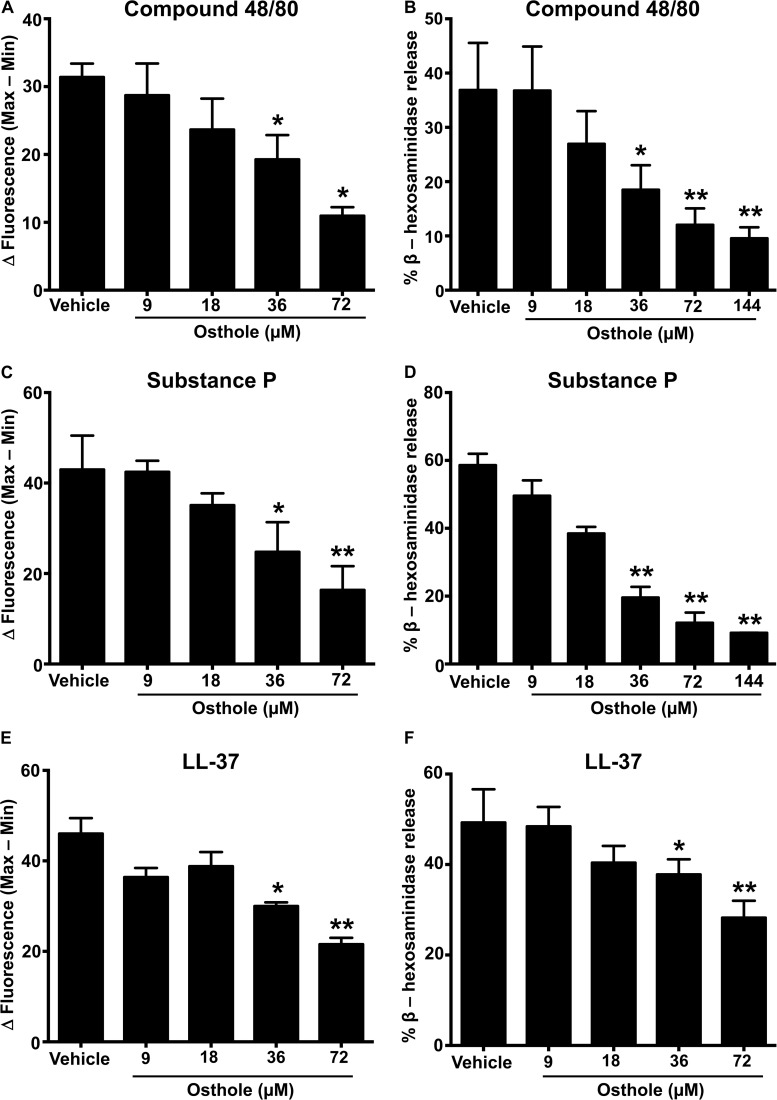
Osthole inhibits MRGPRX2-induced Ca^2+^ mobilization and degranulation in LAD2 mast cells. **(A,C,E)** Intracellular Ca^2+^ mobilization and **(B,D,F)** degranulation in LAD2 human mast cells was determined following pre-incubation with vehicle (0.2% DMSO in PBS) or varying concentrations of osthole for 30 min. Cells were stimulated with the MRGPRX2 agonist **(A)** compound 48/80 (100 ng/ml), **(C)** substance P (300 nM) or **(E)** LL-37 (3 μM) and changes in fluorescence intensities were recorded for 90 s. Data are plotted as the change in fluorescence intensity [minimum (Min) subtracted from maximum (Max) value] measurements. Vehicle- or osthole-treated cells were exposed to **(B)** compound 48/80, **(D)** substance P or **(E)** LL-37 and degranulation was quantified by β-hexosaminidase release. Values are plotted as percentages of total cell lysate β-hexosaminidase content. Data shown are mean ± SE of three independent experiments. Statistical significance was determined by unpaired Student’s *t*-test with values compared between the osthole- and vehicle-treated groups *p < 0.05 and **p < 0.01.

MRGPRX2 is activated by several ligands such as compound 48/80 (3, 43), the neuropeptide substance P (8, 36) and the cathelicidin LL-37 (37), and these ligands induce potent Ca^2+^ mobilization and mast cell degranulation. To determine the effects of osthole on the activation of human mast cells, we pretreated LAD2 cells with varying concentrations of osthole, stimulated them with compound 48/80 (Figures 1A,B), substance P (Figures 1C,D) or LL-37 (Figures 1E,F) and measured the Ca^2+^ mobilization and degranulation response. Importantly, osthole did not show any cytotoxicity at the concentrations used for these experiments. The LC50 (lethal concentration 50, the concentration of osthole which is lethal to 50% of the cells) for LAD2 cells and RBL-2H3 mast cells were ∼200 μM and 400 μM, respectively as determined by cell viability (using trypan blue) and MTT assays (Supplementary Figure 1). Osthole by itself did not induce intracellular Ca^2+^ mobilization or degranulation in LAD2 cells (data not shown). However, a significant dose-dependent reduction in Ca^2+^ mobilization (as measured by the change in fluorescence intensity of cells labeled with a Ca^2+^ sensitive dye) (Figure 1A and Supplementary Figure 2A) and degranulation (as measured by the release of β-hexoseaminidase) (Figure 1B) to compound 48/80 was observed following exposure to osthole. Similar results were obtained when the LAD2 cells were stimulated with other MRGPRX2 ligands such as substance P and LL-37 (Figures 1C–F and Supplementary Figures 2B,C).

**FIGURE 2:**
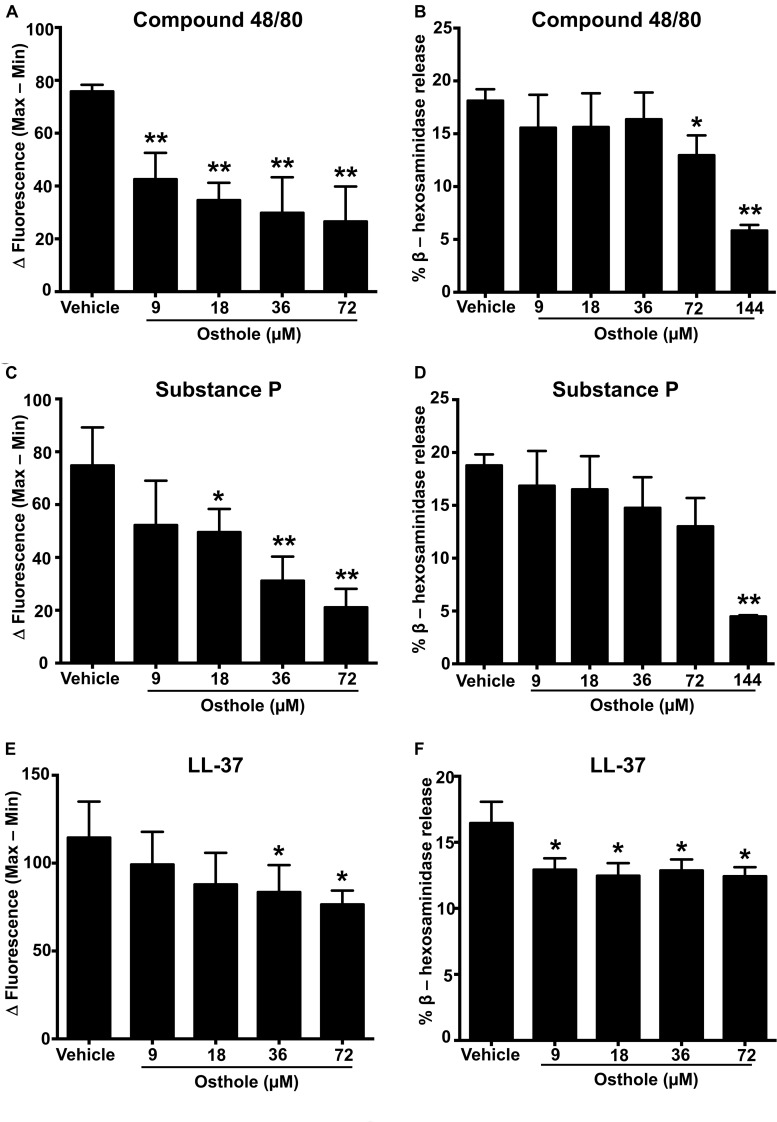
Ca^2+^ mobilization and degranulation induced by MRGPRX2 ligands is reduced by osthole in RBL-2H3 cells stably expressing MRGPRX2. RBL-2H3 cells stably expressing the MRGPRX2 receptor were treated with vehicle (0.2% DMSO in PBS) or osthole (indicated concentrations) for 30 min. **(A,C,E)** Ca^2+^ mobilization and **(B,D,F)** degranulation assays were performed following incubation with **(A,B)** compound 48/80 (100 ng/ml), **(C,D)** substance P (300 nM) or **(E,F)** LL-37 (3 μM). Results shown are mean ± SE of three independent experiments. Statistical significance was determined by unpaired Student’s *t*-test and values from the osthole-treated group was compared with the control vehicle group *p < 0.05 and **p < 0.01.

A recent study identified a synthetic ligand (*R*)-ZINC-3573 as a potent selective agonist for MRGPRX2 (44). To determine if osthole specifically inhibited MRGPRX2 response induced by (*R*)-ZINC-3573, we exposed LAD2 cells to different concentrations of the ligand in the presence of osthole and assessed for Ca^2+^ mobilization and degranulation response. Our data demonstrate that both Ca^2+^ mobilization and degranulation to (*R*)-ZINC-3573 was substantially reduced in the presence of osthole (Supplementary Figures 3A,B). Unlike LAD2 cells, RBL-2H3 cells lack the MRGPRX2 receptor and hence are not activated by the MRGPRX2 ligands. But when MRGPRX2 is expressed in this cell line, the cells degranulate in response to compound 48/80 (43), substance P (8), and LL-37 (37). To determine whether the observed effects of osthole in attenuating mast cell Ca^2+^ mobilization and degranulation to compound 40/80, substance P and LL-37 are specific to the MRGPRX2 receptor, we exposed MRGPRX2 transfected RBL-2H3 cells to varying concentrations of osthole and performed Ca^2+^ mobilization and degranulation experiments. As expected, compound 48/80 (Figures 2A,B and Supplementary Figure 2D), substance P (Figures 2C,D and Supplementary Figure 2E) and LL-37 (Figures 2E,F and Supplementary Figure 2F) induced substantial Ca^2+^ mobilization and degranulation in RBL-2H3 cells expressing MRGPRX2. However, this response was significantly inhibited by osthole in a dose-dependent fashion (Figures 2A–F).

### Osthole Inhibits Chemokine/Cytokine Production and Mitogen-Activated Protein (MAP) Kinase Activation Following MRGPRX2 Stimulation in Mast Cells

**FIGURE 3:**
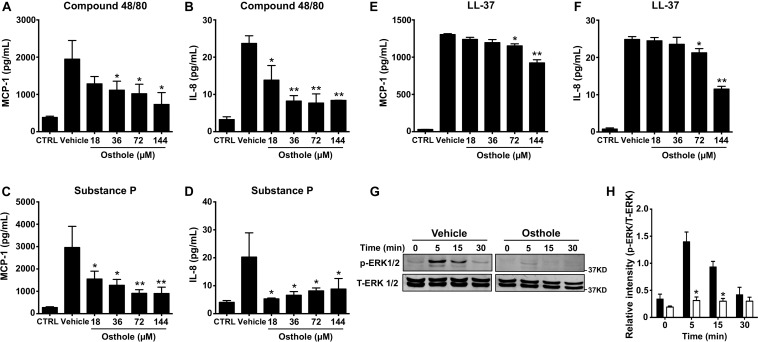
Osthole suppresses cytokine/chemokine production and ERK1/2 activation in LAD2 cells. **(A–F)** LAD2 cells (0.15 × 10^6^ cells/well) were exposed to vehicle (0.2% DMSO in PBS) or different concentrations of osthole for 30 min, and later exposed to media (CTRL), **(A,B)** compound 48/80 (100 ng/ml) or **(C,D)** substance P (300 nM) or **(E,F)** LL-37 (5 μM) for 6 h. Supernatants were collected and analyzed for **(A,C,E)** MCP-1 and **(B,D,F)** IL-8 by ELISA. Data shown are mean ± SE of three independent experiments performed in triplicates. Statistical analysis was done using unpaired Student’s *t*-test by comparing the control vehicle- and osthole-treated groups *p < 0.05 and **p < 0.01. **(G,H)** LAD2 cells were treated with vehicle (0.2% DMSO in PBS) or osthole (72 μM) and then exposed substance P (300 nM) for different time intervals. **(G)** Western blotting was performed to detect phosphorylated ERK1/2 (p-ERK1/2) protein. The blots were stripped and reprobed with total ERK1/2 (T-ERK1/2) antibodies. Images of representative blots are shown. **(H)** Bar graph shows relative intensities of bands for ERK1/2. p-ERK1/2 levels were normalized to T-ERK1/2 expression. Data from three independent experiments are shown mean ± SE. Statistical significance was determined by unpaired Student’s *t*-test *p < 0.05.

Mast cell activation comprises of early events that include intracellular Ca^2+^ mobilization and degranulation and a delayed phase that ultimately results in inflammatory chemokine/cytokine production and their release. To test whether osthole regulated the delayed response of mast cell activation, we exposed LAD2 cells to osthole and assessed for chemokine and cytokine generation by ELISA. We specifically chose to examine the production of MCP-1 and IL-8 since LAD2 cells release these effectors upon on MRGPRX2 stimulation (45). LAD2 cells produced both MCP-1 and IL-8 on stimulation with compound 48/80 (Figures 3A,B), substance P (Figures 3C,D) and LL-37 (Figures 3E,F). Interestingly, osthole treatment inhibited the release of these inflammatory mediators. Overall, our data demonstrate that osthole attenuates both the early and delayed stages of mast cell activation via MRGPRX2.

Previous reports have established that MRGPRX2-induced mast cell activation results in downstream signaling events that activate the MAP kinase ERK1/2 (46). This signaling pathway ultimately regulate mast cell degranulation and cytokine production (47, 48). Since osthole attenuated the functional responses of mast cells, we hypothesized that it likely affects upstream signaling events such as MAP kinase activation. Accordingly, we exposed LAD2 cells to substance P for different time intervals in the presence of osthole and analyzed MAP kinase activation by Western blotting (Figures 3G,H). We chose to use substance P for these studies since this ligand induced the strongest activation of the MAP kinase pathway as compared to other MRGPRX2 ligands (compound 48/80 and LL-37, data not shown). Robust phosphorylation of the MAP kinase ERK1/2 was evident within 5–15 min following exposure to substance P (Figures 3G,H). Notably, ERK1/2 phosphorylation was significantly inhibited by treatment with osthole at the early (5- and 15-min) time points (Figures 3G,H). Taken together, our data suggests that following MRGPRX2 activation, osthole attenuates Ca^2+^ mobilization and downstream MAP kinase signaling events; thus, diminishing functional responses such as degranulation and cytokine/chemokine production.

### MRGPRX2-Mediated Degranulation of Primary Skin-Derived Human Mast Cells Is Reduced in the Presence of Osthole

**FIGURE 4:**
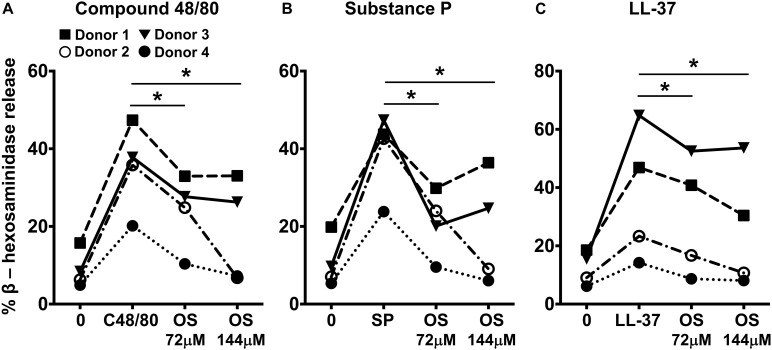
Osthole attenuates MRGPRX2-mediated degranulation in human skin mast cells. **(A–C)** Human skin-derived mast cells were pre-treated with osthole (OS, 72 and 144 μM) and exposed to **(A)** compound 48/80 (C48/80, 100 ng/ml), **(B)** substance P (SP, 300 nM) or **(C)** LL-37 (3 μM). Graphs show degranulation of mast cells as estimated by β-hexosaminidase release in supernatants. Data shown are from 4 different human donors with each symbol representing a single donor. Statistical significance was determined by paired Student’s *t*-test *p < 0.05 and **p < 0.01.

Primary human mast cells derived from the peripheral blood as well as human skin mast cells express MRGPRX2 and respond to MRGPRX2 agonists (5, 15, 37). To confirm the biological relevance of our studies obtained with LAD2 cells, we cultured mast cells isolated from the human skin of 4 different donors and then exposed these cells to osthole and assessed their degranulation response to compound 48/80, substance P and LL-37. Consistent with our data from LAD2 cells, osthole treatment significantly reduced the degranulation of human skin mast cells to the MRGPRX2 agonists (Figures 4A–C).

### Osthole Administration Attenuates Mast Cell-Dependent Inflammation *in vivo*

**FIGURE 5:**
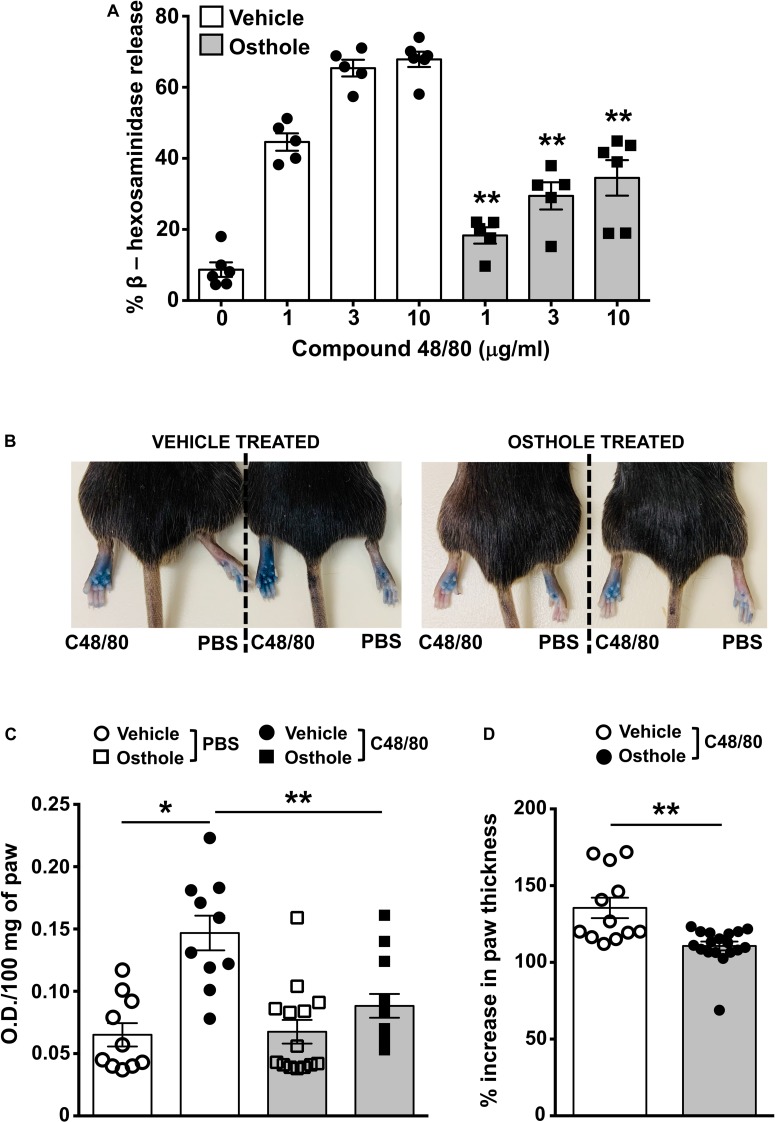
Osthole reduces paw edema to compound 48/80 *in vivo*. **(A)** Mouse peritoneal cells were exposed to vehicle (0.2% DMSO in PBS) or osthole (72 μM) for 30 min and stimulated with the indicated concentrations of compound 48/80. Graph shows degranulation of mast cells as estimated by β-hexosaminidase release in supernatants. **(B–D)** Vehicle- (0.2% DMSO in PBS) or osthole- (100 mg/kg) treated C57BL/6 mice were exposed to compound 48/80, (C48/80, left paw) or PBS (right paw) and Evans blue dye was injected i.v., Mice were culled 30 min later. **(B)** Representative pictures of mice with dye leakage in the paws are shown. **(C)** The paws were excised and weighed; the dye was extracted and absorbance of the supernatant was measured at 650 nm. **(D)** Bar graph shows the change in paw thickness (%) of mice following compound 48/80 injection. Data shown are mean ± SE from three experiments (a total of *n* = 5–13 mice/group). Statistical significance was determined by unpaired Student’s *t*-test *p < 0.05 and **p < 0.01.

Mice express an orthologous receptor to the human MRGPRX2, termed MrgprB2 (3). Similar to its human counterpart, the mouse MrgprB2 receptor is activated by compound 48/80, substance P and LL-37, and induces pseudo-allergic responses. To determine if osthole regulates MrgprB2 responses in mouse mast cells, we performed degranulation assays with mouse peritoneal lavage cells (that have mast cells) and stimulated them with compound 48/80 in the presence or absence of osthole. Consistent with prior studies (3, 49), compound 48/80 induced dose-dependent degranulation in peritoneal cells, which was significantly reduced in the presence of osthole (Figure 5A). Our next goal was to test the role of osthole in regulating mast cell MrgprB2 responses *in vivo*. We adopted a previously described model of compound 48/80-induced paw edema that is dependent on MrgprB2 expression on mouse mast cells (3). Control vehicle “-” or osthole “-” treated mice were injected with PBS or compound 48/80 in their right and left hind paws, respectively, and vascular permeability (an indicator of mast cell degranulation) was assessed following i.v. injection of the Evans blue dye. We observed increased dye extravasation (indicative of vascular leakage) in the paws treated with compound 48/80, as compared to control PBS-treated paws (Figures 5B,C). This response, however, was significantly reduced in the osthole-treated cohort. In addition, we measured paw thickness before and after compound 48/80 administration. Consistent with the vascular permeability data, osthole significantly attenuated paw thickness following compound 48/80 injection (Figure 5D).

**FIGURE 6:**
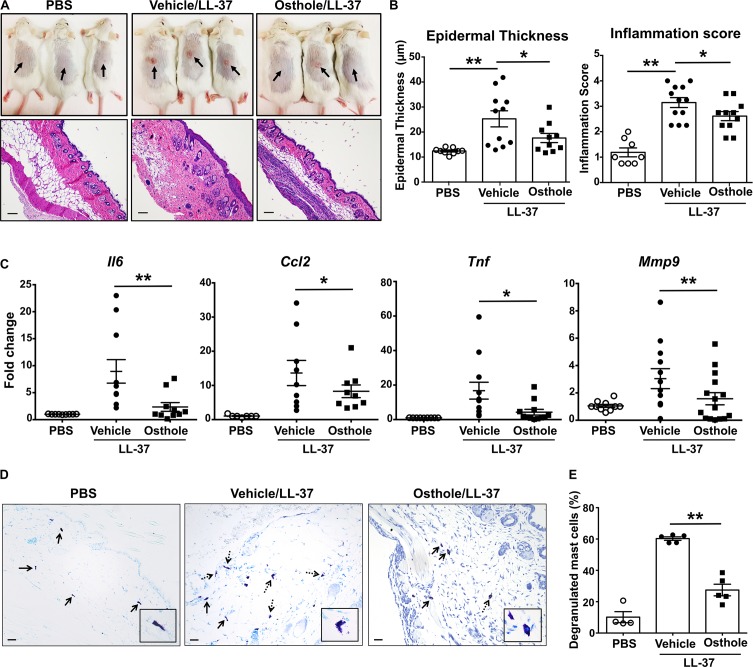
Osthole prevents the development of LL-37-induced rosacea in mice. Vehicle- (0.2% DMSO in PBS, Vehicle/LL-37) or osthole- (Osthole/LL-37) treated Balb/c mice were injected with LL-37 into the dorsal skin (arrows) twice daily for 2 consecutive days. Mice that only received PBS on the dorsal skin were used as control. **(A)** Representative pictures of the dorsal skin (top panels) and H&E stained skin sections (bottom panels) of mice from different cohorts are shown. Scale bar = 100 μm. **(B)** Graphs represent inflammation scores and epidermal thickness of the H&E stained skin sections. **(C)** mRNA expression of selected gene targets from the excised skin was analyzed by real-time PCR. Values are plotted as fold change (2^– ΔΔCt^) normalized to GAPDH levels. **(D)** The paraffin embedded skin sections from different cohorts of mice were stained with toludine blue to detect mast cells. Representiative pictures of the skin sections are shown. Bold arrows indicate intact mast cells whereas dotted arrows represent degranulated mast cells. The inset figure is an enlarged image of the mast cell(s) shown in the pictures. Scale bar = 100 μm. **(E)** Graph shows the percentage of degranulated mast cells in the skin tissue of different cohorts of mice. Data are mean ± SE. from *n* = 4–15 mice/group. Statistical significance was determined by unpaired Student’s *t*-test comparing the vehicle vs. osthole treated groups *p < 0.05 and **p < 0.01.

We next investigated the effects of osthole in a more severe skin inflammation model of pseudo-allergic rosacea. The cathelicidin LL-37 is elevated in the skin tissues of human patients with rosacea, and consequently, it has been used for inducing the pathogenesis of experimental rosacea in rodents (50, 51). Specifically, Muto et al., (15) showed that the LL-37 injections in the skin causes rosacea like symptoms in mice that are dependent on the presence of mast cells. LL-37-induced Ca^2+^ mobilization and degranulation were attenuated by osthole in our experiments with human mast cells (Figures 1E,F, 4C). To directly test whether osthole inhibited mouse mast cell response *in vivo*, we pretreated mice with vehicle or osthole, followed by LL-37 administration in the dorsal skin. After 72 h, skin reddening and inflammation were evident in the LL-37-treated mice, which was greatly reduced in the osthole-treated group (Figure 6A, top panels). Histological analysis of the skin tissues showed less cellular infiltration in the skin of osthole-treated mice (Figure 6A, bottom panels), which is consistent with the significant reduction in the inflammation score and epidermal thickness (Figure 6B) as compared to the vehicle-treated group. Moreover, RNA analysis of inflammatory markers also corroborated with the attenuated skin inflammation in the osthole-treated cohort. Specifically, mRNA levels of mast cell inflammatory mediators such as CCL2, IL-6, TNFα and MMP9 were significantly decreased in the presence of osthole (Figure 6C). To directly test if osthole affected mast cell responses in this model, we enumerated the numbers of degranulated and non-degranulated mast cells in the skin tissues of mice. Degranulated mast cells showed reduced toluidine blue staining intensity and/or dispersed cytoplasmic granules whereas non-degranulated cells were intensely stained and the cytoplasmic granules were not distinctly visible. While there was no difference in the total numbers of mast cells between the vehicle and the osthole-treated cohorts of mice (data not shown), the percentage of degranulated mast cells was significantly reduced in the osthole-treated mice as compared to the control vehicle-treated group (Figures 6D,E). These data thus demonstrate that osthole significantly reduces mast cell-induced inflammation associated with pseudo-allergic reactions *in vivo*.

### Molecular Docking Studies of Osthole With MRGPRX2

**FIGURE 7:**
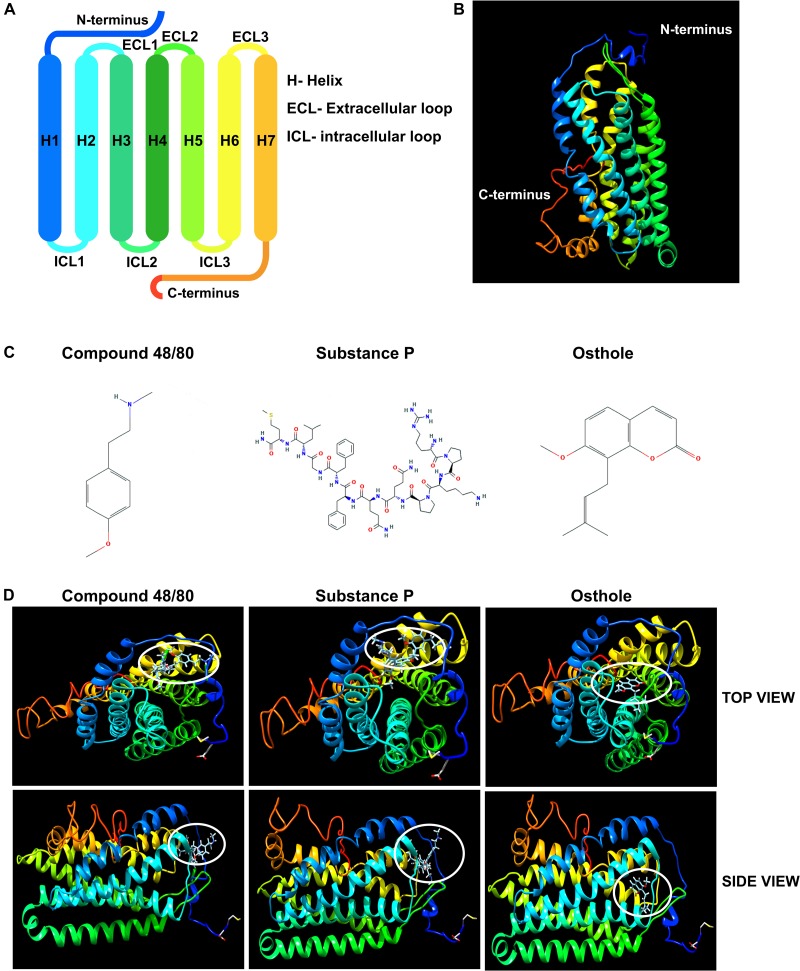
Molecular docking studies of osthole and MRGPRX2. **(A)** Labeled schematic of the MRGPRX2 receptor including the seven transmembrane helices (H), extracellular (ECL) and intracellular (ICL) loops, and the N- and C-termini is shown. **(B)** Predicted molecular model of native MRGPRX2 and **(C)** chemical structures of compound 48/80, substance P and osthole are shown. **(D)** Top view and side view of predicted molecular docking of compound 48/80, substance P and osthole with MRGPRX2 are shown. The region where the ligands or osthole are predicted to interact with the receptor is encircled (white oval).

It has been previously reported that synthetic coumarins can bind to GPCRs and function as antagonists (52). Given that MRGPRX2 is a GPCR (Figures 7A,B), and osthole is a natural coumarin that is structurally similar to compound 48/80 (Figure 7C), it is possible that osthole interacts with MRGPRX2 and competitively inhibits the binding of MRGPRX2 ligands to the receptor resulting in decreased mast cell signaling, degranulation and cytokine production. To investigate this possibility, we performed molecular docking studies with the I-TASSER, Swiss Dock and UCSF Chimera software’s. Both compound 48/80 and substance P were predicted to bind at the N-terminus near the LEU23 and LEU25 residues close to helix 1 and helix 2 (Figure 7D). Conversely, osthole was predicted to bind near PHE93 (between helix 2 and helix 3) in a hydrophobic pocket adjacent to the binding site of compound 48/80 and substance P (Figure 7D). In addition, when osthole is bound to the protein, there appears to be a conformational change in the cytoplasmic tail as well as helix 7 of MRGPRX2. These projected interactions suggest that osthole does not function as a competitive inhibitor but rather behaves as an allosteric inhibitor that induces a structural change in the receptor that results in reduced MRGPRX2 responses.

### Osthole Reduced MRGPRX2 Expression in Human Mast Cells

**FIGURE 8:**
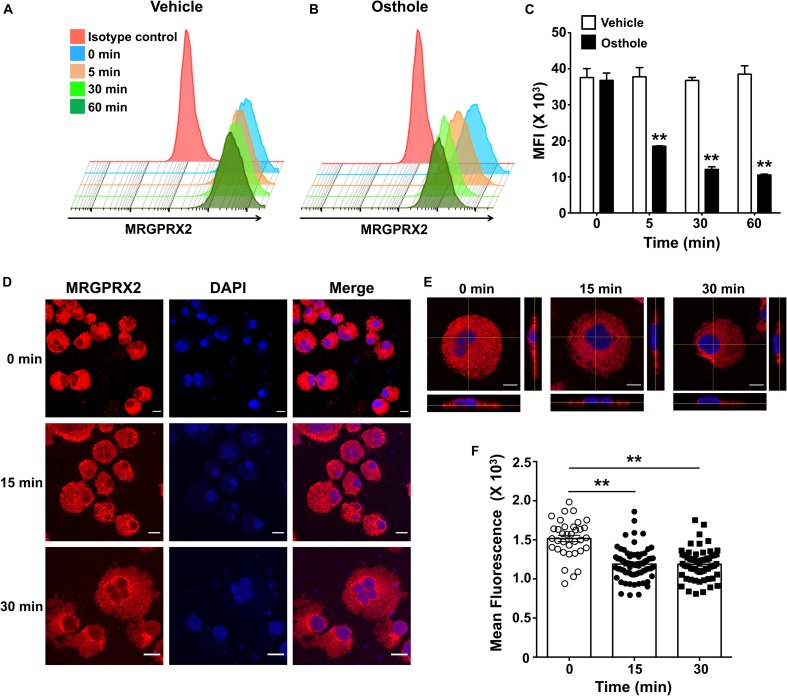
Osthole decreases the expression of MRGPRX2 in LAD2 mast cells. LAD2 cells were exposed to **(A)** vehicle or **(B)** osthole (72 μM) for the indicated time points, washed, stained with PE conjugated MRGPRX2 antibody and analyzed by flow cytometry. Representative histograms of MRGPRX2 surface expression at different time points after vehicle or osthole exposure are shown. **(C)** Bar graph shows the mean fluorescence intensity (MFI) of MRGPRX2 receptor expression following vehicle or osthole treatment. Data are mean ± SE from three experiments. Statistical significance was determined by unpaired Student’s *t*-test comparing the vehicle vs. osthole treated groups *p < 0.05 and **p < 0.01. **(D)** Immunofluorescence staining of MRGPRX2 was performed in LAD2 cells treated with osthole (72 μM) for different time intervals. Representative images of MRGPRX2 expression (red) and nucleus (DAPI) are shown. Scale bar = 10 μm. **(E)** Z-stack confocal microscopy images with cross-sectional views taken at 100 x magnification show localization of MRGPRX2 (red) after osthole exposure. **(F)** Bar graph shows mean fluorescence intensities corresponding to MRGPRX2 expression in LAD2 cells obtained from the confocal experiments. Data represent mean ± SE from three experiments with 30–70 cells analyzed in each group. Statistical analysis was done using unpaired *t*-test **p < 0.01.

The inhibition of mast cell Ca^2+^ mobilization (Figures 1, 2) and MAP kinase signaling (Figure 3) indicates the presence of an upstream mechanism that is altered by osthole. It is thus conceivable that osthole treatment alters MRGPRX2 expression levels resulting in the observed reduction in mast cell response to compound 48/80, substance P and LL-37. To determine if osthole regulated MRGPRX2 levels in mast cells, we exposed LAD2 cells to osthole for varying time intervals and assessed surface receptor expression by flow cytometry. Consistent with previous reports, LAD2 cells expressed high levels of MRGPRX2 on the cell surface; however, osthole treatment resulted in a significant decrease in MRGPRX2 receptor (Figures 8A–C). Strikingly, this reduction was observed as early as 5 min after exposure of cells to osthole (Figure 8C). Several reports have suggested that MRGPRX2 is expressed at both the cell surface as well as in the cytoplasm (5, 6, 53). To determine if osthole also reduced intracellular receptor levels, we exposed LAD2 cells to this natural compound and assessed for MRGPRX2 expression by confocal microscopy. Our data suggests that osthole significantly reduced surface as well as intracellular receptor expression (Figures 8D–F). Specifically, the intensity of MRGPRX2 staining was substantially reduced on the cell surface as well as in the cytoplasm (Figures 8D,E). Interestingly, we observed intense staining of the receptor near and around the cell nucleus after exposure to osthole (Figure 8D, bottom panels). Taken together, osthole reduces MRGPRX2 expression levels in LAD2 mast cells and thereby regulates mast cell responses to compound 48/80, substance P and LL-37.

## Discussion

Since the discovery of MRGPRX2 as a GPCR that is expressed on mast cells, a large body of work by several groups have demonstrated that this receptor is activated by several ligands which include antibiotics, antimicrobial peptides, neuropeptides and anesthetics (4, 53). Additionally, some of the ligands for MRGPRX2, such as the cathelicidin LL-37 and the neuropeptide substance P, are produced by our own cells in response to infection and tissue injury. These ligands can activate tissue-resident mast cells to amplify inflammation, and consequently, mast cells play a pivotal role in mediating the pathophysiology of chronic diseases such as urticaria (5), asthma (6), and rosacea (15), which involve the over-production of the MRGPRX2 ligands. Although these findings highlight the role of the mast cell-MRGPRX2 axis in causing anaphylaxis and inflammation associated with urticaria, asthma and rosacea, there is limited information available on how MRGPRX2 can be targeted to prevent these adverse reactions/diseases in humans. In the current study, we identified a plant compound, osthole that inhibits mast cell-MRGPRX2/MrgprB2 mediated responses *in vitro* and *in vivo*. Specifically, osthole significantly attenuates both the early (Ca^2+^ mobilization and degranulation) and delayed phases (chemokine/cytokine production) of mast cell activation. Furthermore, osthole reduces mast cell-mediated inflammatory responses in two different animal models: (1) an anaphylactic model of paw edema induced by compound 48/80 and (2) a chronic skin inflammatory model of experimental rosacea. Given that mast cell MRGPRX2 plays a pivotal role in mediating pseudo-allergic reactions to several FDA-approved drugs and chronic inflammation associated with asthma, urticaria and rosacea, our studies provide a strong rationale for testing osthole as a novel treatment option for these conditions.

Osthole is a natural coumarin present in the medicinal plant *Cnidium monnieri*. Because osthole demonstrates significant protective effects in animal models of hepatic diseases (30, 31), cancer (54), diabetes (27, 28), osteoarthritis (55), cardiovascular (56, 57) and allergic diseases (18, 34), it is a promising natural lead compound for drug discovery. Herein we demonstrate that osthole inhibits mast cell activation via MRGPRX2. A dose-dependent reduction in mast cell response in the presence of osthole was observed in *in vitro* assays; however, the inhibition was significant only when the cells were exposed to high concentrations of osthole (72 and 144 μM). Even though these concentrations do not induce significant cytotoxicity in human and rat mast cells (Supplementary Figure 1), it is possible that these doses may be toxic to other cell types. This raises a potential concern regarding the therapeutic value and potential toxic off target effects of this natural compound. Therefore, alternative strategies need be developed if this compound is to be approved for use in humans. One such option would be chemically modify osthole and cage it into a polymer scaffold for targeted release only to the desired sites/tissues.

Although osthole is a low molecular weight organic compound, it exhibits low water solubility that reduces its bioavailability and results in decreased absorption (58). Therefore, it is important to enhance the bioavailability of osthole for it to function optimally as a lead compound. As such, the introduction of a more polar group, like a hetero atom or a heterocyclic aromatic ring into the core structure of osthole, will probably improve its bioactivity and physiochemical properties. Another approach will be to encapsulate osthole into a nanoparticle or complex it with cyclodextrins. While previous studies have shown that these methods do indeed enhance the absorption of osthole (59–61), it is also necessary to determine the functional implications of these modifications. In the current study, we demonstrate that osthole inhibits MRGPRX2-mediated mast cell responses such as Ca^2+^ mobilization, degranulation and cytokine/chemokine production. Whether adding an aromatic ring to osthole or caging this molecule in a nanoparticle will preserve its inhibitory effect on mast cell responses *in vitro* and *in vivo*, needs to be validated. Designing synthetic derivatives of osthole that will demonstrate enhanced bioavailability, reduced toxicity while also retaining the functions of the native molecule to attenuate mast cell activation through MRGPRX2, will be the subject of future investigations in our laboratory.

Osthole significantly reduced the Ca^2+^ mobilization, degranulation and cytokine/chemokine production induced by compound 48/80 substance P and LL-37 (Figures 1–3). Interestingly, it did not seem to inhibit LL-37 response as much as it decreased compound 48/80 and substance P induced activation of mast cells. LL-37 is a peptide fragment containing 37 amino acids whereas compound 48/80 and substance P are structurally much smaller (Figure 7). It is possible that LL-37 interacts with MRGPRX2 at a different site as compared to compound 48/80 and substance P. Since LL-37 is larger and bulkier, it may also interact with the receptor at multiple sites. While we were not able to verify these contentions in our modeling studies due to the limitations posed by the analysis software, these differences may actually account for the observed differential inhibitory effects of osthole on the MRGPRX2 ligands tested in the study.

McNeil et al., (3) reported that MrgprB2 is the mouse receptor that is analogous to the human MRGPRX2. MrgprB2 is activated by the same ligands including compound 48/80, substance P and LL-37. Osthole inhibited degranulation of peritoneal cells to compound 48/80 suggesting that this coumarin also regulated MrgprB2 response in mouse mast cells. Consistent with this observation, we demonstrate that compound 48/80-induced paw edema and rosacea caused by LL-37 injections in mice were reduced by osthole administration. These data suggest that osthole inhibits mouse mast cell MrgprB2 response and attenuates inflammation in animal models. Because of its short duration and acute nature, the paw edema model represents the immediate phase of mast cell response, i.e., degranulation. The accumulation of fluids in the paw tissue is due to histamine released by mast cells following degranulation, which causes vasodilation and vascular leakage. Thus, the observed decrease in tissue edema following osthole administration is likely through inhibition of mast cell activation. We further demonstrate that osthole treatment reduces mast cell Ca^2+^ mobilization, degranulation and cytokine/chemokine to LL-37 *in vitro*. Accordingly, the pathology of LL-37-induced rosacea was reduced by osthole *in vivo*. Specifically, we observed attenuation of skin inflammation and a significant reduction in expression of mast cell mediators such as CCL2, IL-6, TNFα and MMP9 in the presence of osthole. These data suggest that mast cell MrgprB2 activation is debilitated by osthole resulting in amelioration of rosacea pathogenesis. It also possible that in addition to inhibiting the mast cell response, osthole also affects the inflammatory response of other immune cells involved in rosacea pathology such as T cells and dendritic cells. Future studies will determine the effect of osthole on inflammatory responses by other immune cells.

All of the MRGPRX2 ligands used in the current study induced substantial and sustained Ca^2+^ mobilization response in LAD2 mast cells. While degranulation and cytokine production are in part dependent on intracellular signaling events, such as Ca^2+^ mobilization, however, other mechanisms do exist that can cause mast cell activation. Notably, osthole significantly reduced intracellular Ca^2+^ mobilization induced by the MRGPRX2 ligands. Several previous studies have suggested that osthole can block Ca^2+^ channels to affect cellular responses (62–64). For example, a recent study by Su et al., (62) showed that osthole can inhibit neuropathic pain behaviors through inhibition of T- and L-type Ca^2+^ currents in nociceptive DRG neurons. Additionally, osthole blocks TRPV channels to prevent pruritis and dermatitis in animal models (63, 64). While it is still unclear whether MRGPRX2 responses in mast cells function via the T- and L-type or TRPV channels, it is plausible that osthole inhibits mast cell activation by blocking these channels resulting in reduced downstream responses such as degranulation and cytokine production. Intracellular signaling events activated by MRGPRX2 are not well characterized; although, the MAP kinase pathway has been shown to be activated in previous studies (45, 46). Our results demonstrated the phosphorylation of ERK1/2 was significantly reduced in the presence of osthole. These results prompted us to examine whether osthole regulated upstream events that resulted in decreased activation of the MAP kinase pathway. Previous studies have demonstrated that coumarins can interact with GPCRs and serve as antagonists and inverse agonists. For e.g., a study conducted by Behrenswerth et al., (52) showed that 5-substituted 3-benzylcourmain derivatives bind to the cannabinoid receptors CB1 and CB2 and function as antagonists for these receptors. To determine whether a natural coumarin such as osthole also regulates MRGPRX2 activation via a similar mechanism, we performed molecular docking studies. Osthole was predicted to bind to the receptor, but this interaction seemed to occur at a different site when compared to the MRGPRX2 ligands, compound 48/80 or substance P. Osthole may thus regulate MRGPRX2 response via allosteric modulation of the receptor rather than competitively inhibiting the ligand interaction. An interesting finding in the current study is that osthole regulates both surface as well as intracellular expression levels of the MRGPRX2 receptor in LAD2 mast cells thus providing another possible mechanism through which osthole inhibits MRGPRX2 responses; reduced receptor expression results in attenuated mast cell activation. Strikingly, we observed intense staining of MRGPRX2 near the perinuclear region in cells treated with osthole. This data suggests that osthole likely blocked the secretion of the receptor from the endoplasmic reticulum and/or the Golgi into the cytoplasm.

Currently, there is no direct evidence that osthole regulates IgE-dependent responses in mast cells. However, a study by Liu et al., (65) showed that a coumarin inhibited IgE-induced mast cell degranulation *in vitro* and allergic responses *in vivo*. Therefore, it is reasonable to speculate that osthole can also inhibit IgE/antigen-induced mast cell activation and thus suppress allergic responses. Indeed, a recent report showed that osthole attenuated allergic asthma in a murine model (34). Specifically, airway inflammation, IgE levels and Th2 cytokines were reduced in the presence of osthole. Since mast cells are important mediators of asthma *in vivo*, it is possible that osthole reduced mast cell activation in this model resulting in reduced Th2 responses and lung inflammation. This is highly significant in situations where individuals experience both IgE-dependent and IgE-independent allergic reactions because osthole treatment will result in attenuation of mast cell activation via both MRGPRX2 and IgE receptors.

## Conclusion

In conclusion, our study uncovers the therapeutic potential of osthole in modulating pseudo-allergic inflammatory responses that occur in humans in response to various FDA-approved drugs. A natural plant compound such as osthole that targets MRGPRX2, may thus provide a safer alternative to treat pseudo-allergic conditions over the current allergy therapies available.

## Data Availability Statement

All datasets generated for this study are included in the article/Supplementary Material.

## Ethics Statement

The studies involving human participants were reviewed and approved by Human studies Institutional Review Board (IRB) of the University of South Carolina. The patients/participants provided their written informed consent to participate in this study. The animal study was reviewed and approved by Institutional Animal Care and Use Committee at Michigan State University.

## Author Contributions

BC and AK performed the experiments, interpreted the data, and wrote parts of the manuscript. MS, CJO, CY, and RN performed the experiments and analyzed the data. AC and CAO isolated and cultured the human mast cells from donor skin tissues, and reviewed the manuscript. RD provided help with the flow cytometry and confocal experiments, and wrote parts of the manuscript. HS conceived the study, planned the experiments, and wrote the manuscript.

## Conflict of Interest

The authors declare that the research was conducted in the absence of any commercial or financial relationships that could be construed as a potential conflict of interest.
