# Supplementary figures and images for: Osthole, a Natural Plant Derivative Inhibits MRGPRX2 Induced Mast Cell Responses
Source: Front Immunol. 2020 Apr 24;11:703. doi: 10.3389/fimmu.2020.00703 (PMC7194083; doi:10.3389/fimmu.2020.00703)

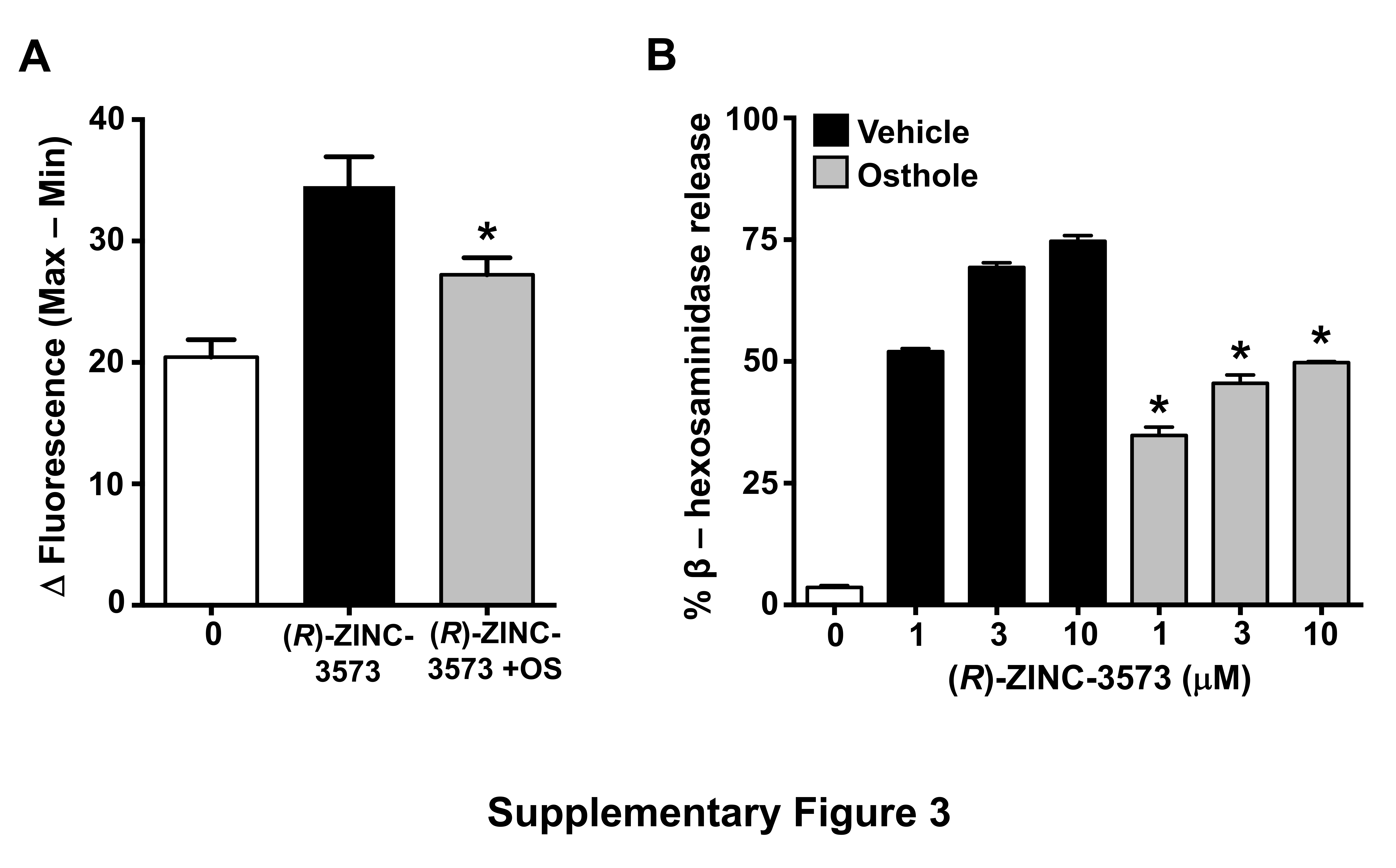

Supplement: Supplementary file 3 [file Image_3.JPEG]
