# Supplementary material for: Osthole, a Natural Plant Derivative Inhibits MRGPRX2 Induced Mast Cell Responses
Source: Front Immunol. 2020 Apr 24;11:703. doi: 10.3389/fimmu.2020.00703 (PMC7194083; doi:10.3389/fimmu.2020.00703)
Supplement: Supplementary file 4 [file Table_1.DOCX]

**Supplementary Figure Legends**

**Figure 1. Cytotoxicity studies of osthole on mast cells.** **(A)** LAD2 cells or **(B)** RBL-2H3 cells were incubated with increasing concentration of osthole for 24 hr. **(A)** Viable LAD2 cells were enumerated using trypan blue staining and a hemocytometer. Line graph shows percent cell survival as compared to vehicle (0.2% DMSO in PBS) treatment. MTT assay was performed to determine the viability of RBL-2H3 cells following osthole treatment. **(B)** Line graph depicts the percent cell survival of RBL-2H3 cells. The results are expressed as the mean ± S.E. values of 3 independent experiments. **(C)** Representative light microscopy images of cells following the MTT assay is shown. Dark areas indicate live cells whereas the lighter areas correlate with dead cells.

**Figure 2. Osthole attenuates Ca^2+^ mobilization to different MRGPRX2 agonists in LAD2 and RBL-2H3 cells stably expressing MRGPRX2.** Intracellular Ca^2+^ mobilization in LAD2 cells (A, B, C) and RBL-2H3 cells expressing MRGPRX2 (D, E, F) was determined following pre-incubation with vehicle (0.2% DMSO in PBS) or osthole (72 μM) for 30 min. Cells were stimulated with **(A, D)** compound 48/80 (100 ng/ml), **(B, E)** substance P (300 nM) or **(C, F)** LL-37 (3 μM) at 20-30 sec (indicated by dashed arrows) and changes in fluorescence intensities were recorded for 80 seconds. Representative traces show the increase in fluorescence intensity following agonist stimulation indicating intracellular Ca^2+^ mobilization.

**Figure 3. Osthole inhibits (*R*)-ZINC-3573-induced Ca^2+^ mobilization and degranulation in LAD2 mast cells. (A)** LAD2 cells were pre-incubated with vehicle (0.2% DMSO in PBS) or osthole (72 μM) for 30 min, labelled with Fluo-8 and stimulated with (*R*)-ZINC-3573 (1 μM). Bar graph shows the change in fluorescence intensity [minimum (Min) subtracted from maximum (Max) value] measurements following agonist treatment. **(B)** Vehicle- or osthole-treated cells were exposed to the indicated concentrations of (*R*)-ZINC-3573 and degranulation was quantified by β-hexosaminidase release. Data shown are mean ± S.E. of 3 independent experiments. Statistical significance was determined by unpaired Student’s *t*-test with values compared between the osthole- and vehicle-treated groups. * p <0.05.
